# IgG Fc Receptors Provide an Alternative Infection Route for Murine Gamma-Herpesvirus-68

**DOI:** 10.1371/journal.pone.0000560

**Published:** 2007-06-27

**Authors:** Gustavo T. Rosa, Laurent Gillet, Christopher M. Smith, Brigitte D. de Lima, Philip G. Stevenson

**Affiliations:** Division of Virology, Department of Pathology, University of Cambridge, Cambridge, United Kingdom; Rockefeller University, United States of America

## Abstract

**Background:**

Herpesviruses can be neutralized in vitro but remain infectious in immune hosts. One difference between these settings is the availability of immunoglobulin Fc receptors. The question therefore arises whether a herpesvirus exposed to apparently neutralizing antibody can still infect Fc receptor^+^ cells.

**Principal Findings:**

Immune sera blocked murine gamma-herpesvirus-68 (MHV-68) infection of fibroblasts, but failed to block and even enhanced its infection of macrophages and dendritic cells. Viral glycoprotein-specific monoclonal antibodies also enhanced infection. MHV-68 appeared to be predominantly latent in macrophages regardless of whether Fc receptors were engaged, but the infection was not abortive and new virus production soon overwhelmed infected cultures. Lytically infected macrophages down-regulated MHC class I-restricted antigen presentation, endocytosis and their response to LPS.

**Conclusions:**

IgG Fc receptors limit the neutralization of gamma-herpesviruses such as MHV-68.

## Introduction

Persistent viruses typically transmit infection by reactivating in immune hosts. Thus, they differ fundamentally from epidemic viruses, whose replication and transmission are blocked by established immunity. The chief defence against epidemic viruses is neutralizing antibody [1], which mainly blocks receptor binding [2]. How persistent viruses evade the same neutralization is not well understood. Some employ antigenic variation [3], but herpesviruses-arguably the most sophisticated of all persistent viruses-do not do so to any significant degree. Herpes virions are shed at low levels, while anti-viral antibody titers are often high [4]. And *in vitro* neutralization is abundantly documented [5]–[8]. However, virus carriers still spread infection. Standard *in vitro* neutralization assays therefore fail to capture some important aspects of infection *in vivo*.

Our understanding of gamma-herpesvirus neutralization has been limited by the narrow species tropisms of Epstein-Barr virus (EBV) and the Kaposi's Sarcoma-associated Herpesvirus (KSHV). Both fail to reproduce their basic pathogenesis in experimental animals and propagate poorly *in vitro*, making related gamma-herpesviruses an important source of information. One of the most experimentally accessible is murine gamma-herpesvirus-68 (MHV-68), a natural, B cell-tropic parasite of yellow-necked mice [9]–[12]. The basic mechanisms of host colonization appear to be quite similar between these viruses. However, their diseases are not-EBV and KSHV cause tumours, whereas MHV-68 pathologies primarily reflect lytic replication [13], [14]. Thus, MHV-68 models normal gamma-herpesvirus biology much better than it does human disease. Distinguishing normal host colonization from disease is important in pathogenesis studies. CD4^+^ T cells [15], [16], CD8^+^ T cells [17] and antibody [16], [18]–[20] can all protect against MHV-68-induced disease, because viral immune evasion operates poorly at high levels of lytic replication [21], [22]. But host immunity fails to block herpesvirus transmission, because evasion dominates at low levels of lytic replication [21], [22].

Our aim with MHV-68 is to understand why established antibody responses do not prevent gamma-herpesvirus transmission. Standard MHV-68 pathogenesis studies have a limited capacity to answer this question as they focus on acute infection; by the time MHV-68-specific antibody is made, virus titers are low. And no MHV-68 transmission model yet exists. Immune sera protect antibody-deficient mice against disease [16], [18]–[20], so antibody can damp down pathological lytic replication. But these studies have not distinguished neutralization from antibody-dependent cytotoxicity. Comparison with Herpes simplex virus [23] would suggest that mainly the latter protects against disease [24]. In contrast, blocking transmission must require neutralization.

It is essential with MHV-68 not only to record *in vivo* phenomena but to identify the mechanisms behind them. Fortunately, MHV-68 offers several advantages for *in vitro* analysis: it is readily propagated and modified, cells with relevant deficiencies can be derived from knockout mice, and monoclonal antibodies can be generated from virus carriers. Our approach has therefore been first to understand as much as possible about neutralization *in vitro*. Immune sera appear to block MHV-68 infection of fibroblasts [25], [26] primarily by blocking cell binding [27]. This raises questions about the robustness of neutralization, as antibody-coated viruses could still potentially infect macrophages and dendritic cells via immunoglobulin Fc receptors (FcRs) [28], [29]. MHV-68 replicates in both these cell types [30], [31]. And FcRs on intraepithelial dendritic cells [32] or epithelial FcRn [33], [34] could allow antibody-coated virions to enter new hosts. We have tested here whether antibody-mediated MHV-68 neutralization applies equally to FcR^+^ and FcR^−^ cells. Our data suggest that FcRs allow otherwise neutralized virions to remain infectious and are therefore likely to compromise infection control.

## Results

### Immune sera fail to block MHV-68 infection of Fc receptor^+^ cells

**Figure 1:**
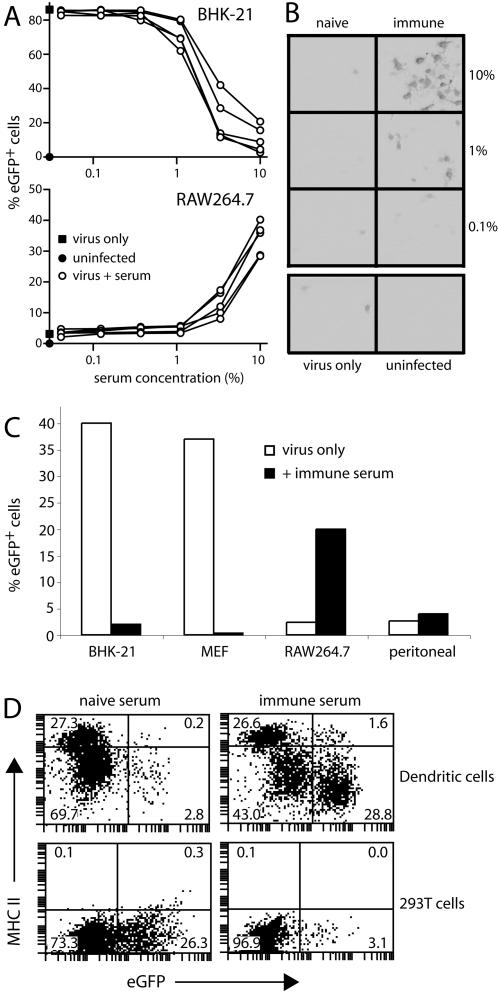
Immune sera enhance MHV-68 infection of Fc receptor^+^ cells. **A.** BAC^+^ MHV-68 virions (4×10^6^PFU/ml) were incubated (1 h, 37°C) with sera from MHV-68-immune C57BL/6 mice. The serum/antibody mixtures were then added to BHK-21 fibroblasts (1PFU/cell) or RAW264.7 macrophages (3PFU/cell). 18h later infection was measured by flow cytometry of viral eGFP expression. Each line corresponds to 1 serum sample, pooled from 2 mice. The data are from 1 of 5 equivalent experiments. **B.** An equivalent experiment using sera from naive or MHV-68-immune BALB/c mice was analyzed by fluoresence microscopy. Each picture shows a 50% confluent culture of RAW264.7 cells. EGFP^+^ cells appear black. **C.** BAC^+^ MHV-68 virions and immune serum (20 µL/3×10^5^PFU) were incubated together (1 h, 37°C) and then divided between BHK-21 cells (0.3PFU/cell), murine embryo fibroblasts (MEF, 0.3PFU/cell), RAW264.7 cells (1PFU/cell) and peritoneal macrophages (1PFU/cell). Viral eGFP expression in each population was determined 18h later by flow cytometry. The data are from 1 of 3 equivalent experiments. CD19^+^ peritoneal cells were always <1% eGFP^+^. **D.** Bone marrow-derived dendritic cells or 293T epithelial cells were infected with MHV-68 (3PFU/cell) that expressed eGFP-tagged gM and had been pre-treated with 1% MHV-68-immune or naive mouse serum. 21 h later, gM-eGFP expression was quantitated by flow cytometry. Dendritic cells were CD11c^+^ and co-stained for MHC class II as shown.

**Figure 2:**
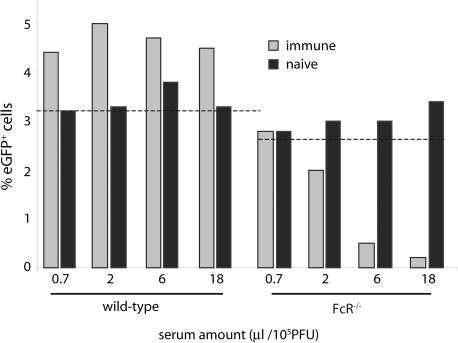
The effect of immune serum on macrophage infection depends on IgG Fc receptors. Wild-type and FceRIγ^−/−^FcγRIIB^−/−^ (FcR^−/−^) bone marrow-derived macrophages were incubated overnight with BAC^+^ MHV-68 (5PFU/cell) that had been preincubated with dilutions of serum from either naive or MHV-68-immune mice, and then analyzed for viral eGFP expression by flow cytometry. The dashed lines show the level of infection with no antibody. The data are from 1 of 3 equivalent experiments.

Immune sera block MHV-68 infection of fibroblasts [25], [26]. In order to test whether this neutralization applied also to FcR^+^ cells, we incubated MHV-68 virions with sera from immune mice and then added the antibody-coated virions to either BHK-21 fibroblasts or RAW264.7 macrophages (Fig. 1). The virus used (BAC^+^) expresses eGFP from a human cytomegalovirus (HCMV) IE1 promoter, so eGFP expression provides a convenient marker of infection [35]. Immune sera inhibited BHK-21 cell infection but enhanced RAW264.7 cell infection (Fig. 1A, 1B). Naive sera had no effect (Fig. 1B), and neither heating immune sera (1 h, 56°C) nor adding exogenous mouse complement to untreated or heat-treated sera altered infection enhancement (data not shown). The enhancement was less obvious for peritoneal macrophages (Fig. 1C). However, all sera (n>20) blocked fibroblast infection and failed to block RAW264.7 cell or peritoneal macrophage infections. Dendritic cell infection, this time measured by eGFP-tagged gM expression rather than HCMV IE1-driven eGFP, was also enhanced rather than blocked by immune serum (Fig. 1D). Immune sera enhanced BAC^+^ MHV-68 infection of wild-type, bone marrow-derived macrophages, but inhibited the infection of IgG FcR-deficient macrophages (Fig. 2). Thus, the failure of antibody to inhibit macrophage infection was due to IgG FcRs, and RAW264.7 cell infection represented a general phenomenon of FcRs undermining virus neutralization.

### Viral glycoprotein-specific monoclonal antibodies (mAbs) can also enhance MHV-68 infection of Fc receptor^+^ cells

**Figure 3:**
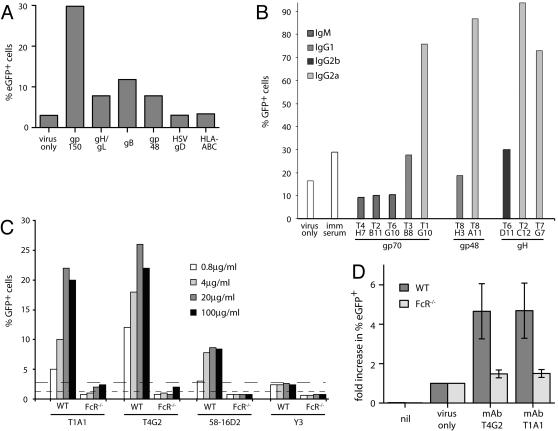
MHV-68 glycoprotein-specific monoclonal antibodies enhance the infection of Fc receptor^+^ cells. **A.** Purified BAC^+^ virions (10^6^PFU/ml) were incubated (1 h, 37°C) either alone (virus only) or with isotyped-matched mAbs (all IgG2a at 10 µg/ml): gp150-T1A1, gH/gL-T2C12, gB-T7H9, gp48-6D10. MAbs LP2 (anti-HSV gD) and W6/32 (Anti-HLA-ABC) provided negative controls. The virion/antibody mixtures were added to RAW264.7 cells (3PFU/cell). Infection was quantitated 18h later by flow cytometry of viral eGFP expression. The data shown are representative of >10 different experiments with at least 4 different mAbs tested for each viral protein. T2C12 is a neutralizing mAb, but 10 µg/ml corresponds to a sub-neutralizing dose for the amount of virus used. **B.** BAC^+^ MHV-68 virions were incubated (1 h, 37°C) with no serum (virus only), 1% MHV-68-immune serum (imm serum), or mAbs of different isotypes as indicated (10 µg/ml) and then added to RAW264.7 cells (5PFU/cell). Viral eGFP expression was quantitated by flow cytometry 18h later. The data are from 1 of 5 equivalent experiments. **C.** Wild-type (WT) and FceRIγ^−/−^FcγRIIB^−/−^ (FcR^−/−^) bone marrow-derived macrophages were incubated overnight with BAC^+^ MHV-68 virions plus mAb dilutions as shown. T1A1 and T4G2 recognize gp150, 58-16D2 recognizes gp60 and Y3 is an H2-K^b^-specific control. The dashed lines show infection with no antibody for WT (coarse dashes) and FcR^−/−^ macrophages (fine dashes). The data are from 1 of 3 equivalent experiments. **D.** Wild-type (WT) or CD16^−/−^CD32^−/−^CD64^−/−^ (FcR^−/−^) bone marrow-derived macrophages were left uninfected (nil) or infected (3PFU/cell) with BAC^+^ virions that had been preincubated or not (virus only) with gp150-specific mAbs as shown (T4G2, T1A1). Infection was quantitated 18 h later by flow cytometric assay of viral eGFP expression. Virus alone typically gave eGFP expression in 1–2% of bone marrow macrophages. The bars show mean±SD results from 3 experiments.

Immune sera comprise complex mixtures of antibody specificities, titers and isotypes. Thus, while they provide a useful measure of the overall host response, different components of that response can be hard to discern. For example, sera might contain both infection inhibiting and infection enhancing antibodies. We therefore tested a range of MHV-68 glycoprotein-specific mAbs for FcR-dependent infection (Fig. 3). Almost all glycoprotein-specific IgG mAbs enhanced RAW264.7 cell infection by BAC^+^ MHV-68 to some degree. Gp150-specific mAbs were the most effective, but some enhancement could be seen even with mAbs specific for the neutralization targets gH/gL [27] and gB [36] (Fig. 3A). Although we did not have sufficient mAbs for comprehensive testing, IgM mAbs tended to inhibit infection and IgG2a mAbs enhanced better than IgG1 or IgG2b (Figure 3B). As with serum-mediated enhancement, mAb-mediated enhancement was much reduced with FcR-deficient macrophages (Fig. 3C, 3D).

### Increased viral eGFP expression correlates with other measures of infection

**Figure 4:**
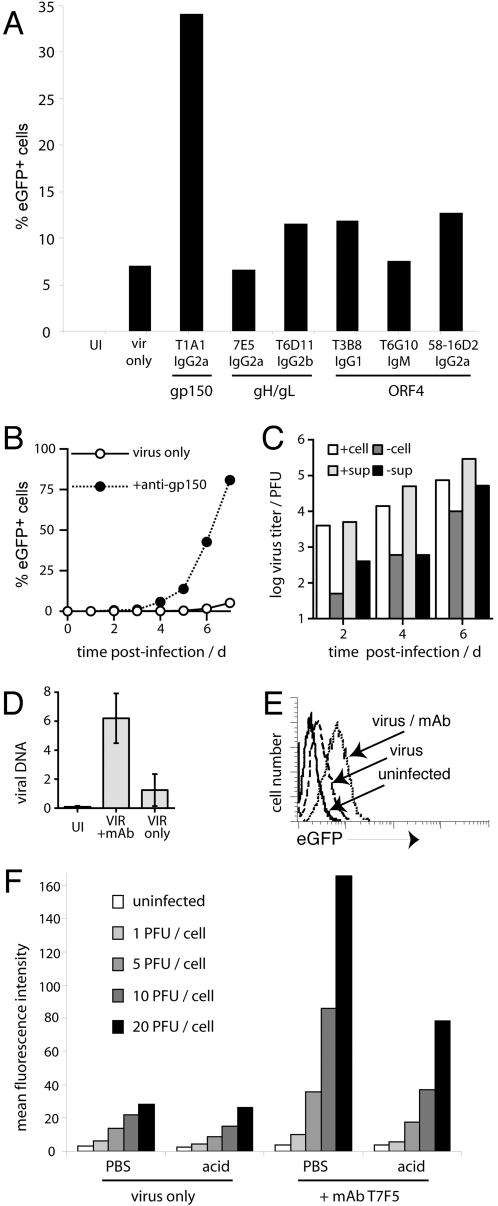
Antibody-dependent infection is productive and correlates with increased cell binding. **A.** MHV-68 expressing eGFP via an IRES inserted downstream of ORF73 was incubated with viral glycoprotein-specific mAbs as shown (100 µg/ml) and then incubated with RAW264.7 cells (5PFU/cell). UI = uninfected; vir only = virus without antibody. 18 h later viral eGFP expression was quantitated by flow cytometry. The data are from 1 of 3 equivalent experiments. **B.** BAC^+^ MHV-68 was incubated with the gp150-specific mAb T4G2 (20 µg/ml) or with medium alone (1 h, 37°C) and then added to RAW264.7 cells (5PFU/cell). The cells were washed with PBS after 2h and then added to a 5-fold excess of uninfected RAW264.7 cells to monitor viral spread. Replicate cultures were analyzed for eGFP expression at the time points indicated. **C.** Replicate samples from the experiment in B, either with (+) or without (−) mAb preincubation for the initial infection, were divided into cell and supernatant (sup) fractions, frozen and thawed, and plaque assayed for infectious virus. The data are from 1 of 2 equivalent experiments. **D.** MHV-68 virions were incubated (1 h, 37°C) with 20 µg/ml mAb T4G2 (VIR+mAb) or with medium alone (VIR only), then added to RAW264.7 cells (8 h, 37°C). The cells were then washed in pH = 3 citrate buffer to remove accessible virus and lysed. Viral DNA content was quantitated by real-time PCR. Each bar shows mean±SD of 4 replicates. The data are from 1 of 2 equivalent experiments. **E.** MHV-68-gM-GFP was pre-incubated with mAb T1A1 (100 µg/10^7^PFU) (dotted line) or with medium only (1 h, 37°C) (dashed line), then added to RAW264.7 cells (5PFU/cell). The cells were washed with PBS after 4 h at 37°C, and assayed for gM-eGFP uptake by flow cytometry. **F.** gM-eGFP MHV-68 was incubated with RAW264.7 cells at different multiplicities with or without the gp150-specific mAb T7F5 (10 µg/ml). 4h later the cells were washed either in PBS or in pH = 3 citrate buffer (acid). gM-eGFP uptake was then assayed by flow cytometry. The data are from 1 of 2 equivalent experiments.

Since the behaviour of an HCMV IE1 promoter in the context of the MHV-68 genome is unknown, we tested whether BAC^+^ eGFP expression in RAW264.7 cells correlated with other markers of infection (Fig. 4). First, preincubating virions with a glycoprotein-specific mAb also gave more eGFP^+^ cells when eGFP expression was driven by an endogenous MHV-68 promoter (Fig. 4A). We used that of ORF73, since it is active in latency as well as in lytic infection [37], [38]. Second, a gp150-specific mAb increased both the dissemination of BAC^+^ eGFP to new cells (Fig. 4B) and BAC^+^ virus replication (Fig. 4C). FcR-dependent infection was therefore productive. Third, real-time PCR showed that pre-incubation with antibody increased the uptake of viral genomes (Fig. 4D) and eGFP-tagged (gM-eGFP) virions (Fig. 4E). Comparing neutral and low pH washes suggested mAbs primarily increased virus binding (Fig. 4F).

### HCMV IE1 promoter activity underestimates macrophage infection

**Figure 5:**
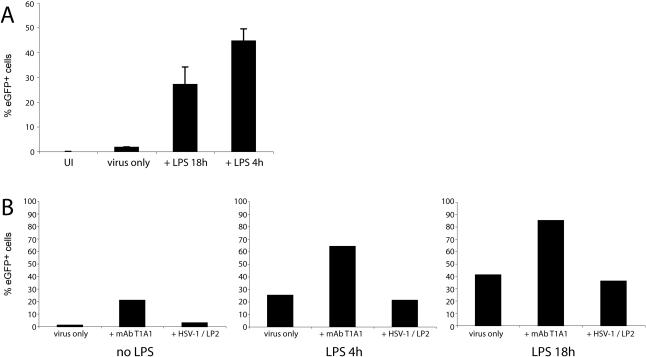
LPS reveals higher levels of RAW264.7 cell infection for both antibody-independent and antibody-dependent infections. **A.** RAW264.7 cells were left uninfected (UI) or incubated overnight with BAC^+^ MHV-68 virions (2PFU/cell). LPS (100 ng/ml) was added to the infected cells for the full 18h, for 4h following infection, or not at all (virus only). Viral eGFP expression was then quantitated by flow cytometry. The bars show mean±SD of 3 replicate cultures. Equivalent results were obtained in 3 repeat experiments with RAW264.7 cells and 3 further experiments with peritoneal macrophages. **B.** RAW264.7 cells were incubated overnight with BAC^+^ MHV-68 virions (2PFU/cell) with no LPS, with LPS for 4h before assay, or with LPS for the whole 18h. There was either no additional treatment (virus only), or the cultures were supplemented with the gp150-specific mAb T1A1 (10 µg/ml) or with LP2-treated HSV-1 virions. LP2, kindly provided by Ms. S.Bell (Division of Virology), is a gD-specific IgG2a neutralizing mAb and was added at concentrations sufficient to ablate HSV-1 infectivity (data not shown). Viral eGFP expression was measured by flow cytometry. The data are from 1 of 2 equivalent experiments.

**Figure 6:**
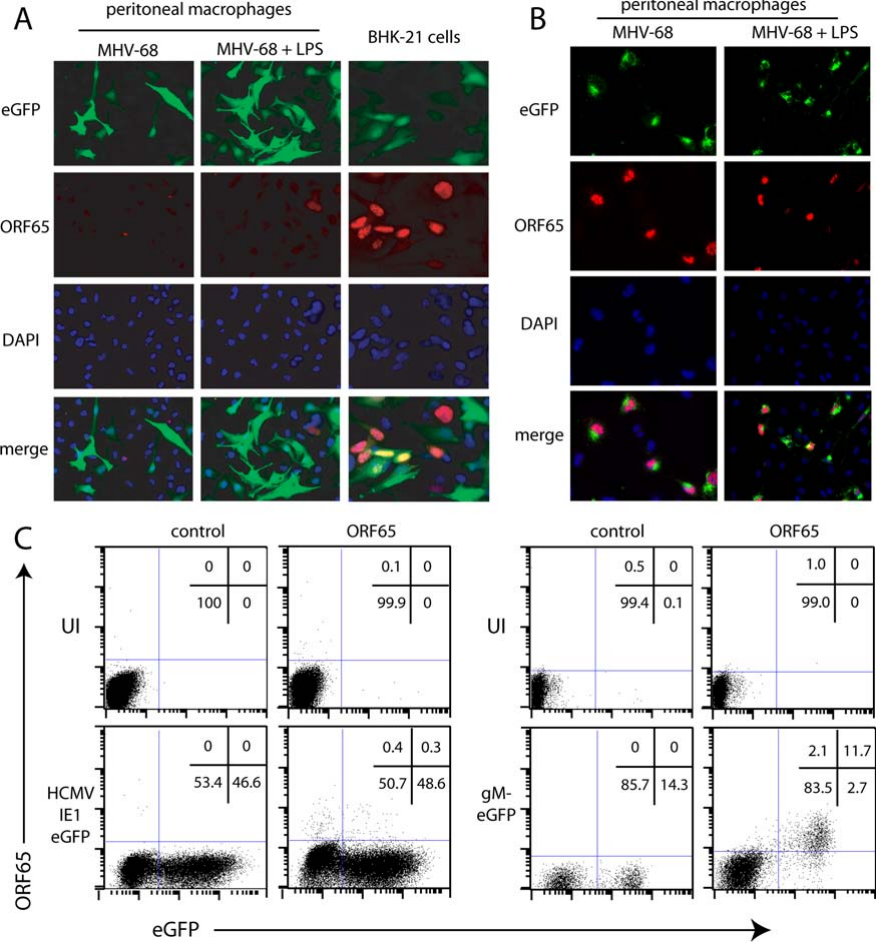
gM-eGFP expression but not HCMV IE1 promoter-driven eGFP expression correlates with ORF65 capsid expression in MHV-68-infected macrophages. **A.** Peritoneal macrophages or BHK-21 fibroblasts were infected with BAC^+^ MHV-68 (2PFU/cell and 0.5PFU/cell respectively). The macrophages were also treated or not with LPS (100 ng/ml). 18h later the cells were fixed in 4% paraformaldehyde and stained with the ORF65-specific mAb 12B8 plus Alexa 568-coupled goat anti-mouse IgG pAb (red). Nuclei were counterstained with DAPI (blue). eGFP was visualized directly (green). The data are from 1 of 3 equivalent experiments, and similar results were obtained with RAW264.7 cells. **B.** Peritoneal macrophages were infected as in **A** but with MHV-68 expressing eGFP-tagged gM, then fixed and stained for ORF65. The data are from 1 of 3 equivalent experiments, and similar results were obtained with RAW264.7 cells. C. RAW264.7 cells were left uninfected (UI) or infected overnight (2PFU/cell) with either BAC^+^ or gM-eGFP MHV-68. The cells were stimulated with LPS (100 ng/ml) for a further 4 h to maximize HCMV IE1 promoter activity, then fixed, permeabilized with 0.1% Tween-20 and stained for ORF65 with mAb 12B8 plus Alexa 633-conjugated goat anti-mouse IgG pAb. Control = secondary antibody only. The percentage of cells in each quadrant is shown. The data are from 1 of 2 equivalent experiments.

Although HCMV IE1 promoter-driven eGFP expression correlated with other measures of infection, it was unclear whether all infected cells were eGFP^+^ and whether eGFP expression correlated mainly with lytic or latent viral gene expression. There is currently no definitive marker of MHV-68 latency. Thus, to test whether some eGFP^−^ cells might also be infected we exploited the fact that the HCMV IE1 promoter is activated by LPS [39] and treated RAW264.7 cells exposed to MHV-68 with LPS (Fig. 5A). The number of eGFP^+^ cells increased considerably. This was true even when LPS was added after removing the input virus, so it did not act by increasing infection. Instead, the HCMV IE1 promoter was active in only a minority of MHV-68-infected RAW264.7 cells. The same was true of peritoneal macrophages (see Fig. 6A). LPS had no effect on eGFP expression from the MHV-68 ORF73 promoter (data not shown).

This result raised the possibility that increased eGFP expression by antibody reflected greater reporter gene expression as well as greater infection. We therefore tested the effect of antibody on BAC^+^ eGFP expression in RAW264.7 cells with and without LPS (Fig. 5B). LPS increased eGFP expression in all samples, but the increase was similar for each. And adding neutralized HSV-1/IgG2a complexes to MHV-68-infected RAW264.7 cells did not increase their eGFP expression, arguing that Fc receptor engagement had little effect on the HCMV IE1 promoter. Thus, although HCMV IE1 promoter activity underestimated the total number of infected macrophages, it gave a good relative indication of infection in different populations, and this was increased by antibody. The fold increase in BAC^+^ eGFP expression clearly depends in part on the basal level, so the fold increase in RAW264.7 cell infection was lower when LPS was included, in fact closer to that of primary macrophages. The difference in enhancement between these cell types may reflect that the HCMV IE1 promoter is more active in explanted primary macrophages. Again, the key point is not enhancement, but that in the presence of FcRs immune sera failed to neutralize.

### Most macrophages showing HCMV IE1 promoter activity are not lytically infected

Our second question was whether BAC eGFP^+^ macrophages predominantly supported lytic or latent infection. We answered this by immunostaining infected peritoneal macrophages for the ORF65 capsid component (Fig. 6). Perinuclear capsids were observed in both eGFP^+^ and eGFP^−^ macrophages, consistent with endocytic virion uptake [36], but very few of either showed the strong nuclear staining typical of new capsid expression, as was seen in lytically infected BHK-21 cells (Fig. 6A, 6C). Thus, most BAC eGFP^+^ macrophages appeared to be latently infected. This was also true of RAW264.7 cells, consistent with RAW264.7 cell cultures infected with BAC^+^ MHV-68 and >50% eGFP^+^ containing much less infectious virus and surviving much longer than equivalent BHK-21 cell cultures (data not shown).

Even BHK-21 cells showed a limited correlation between BAC eGFP expression and nuclear capsid staining (Fig. 6A). Thus, the HCMV IE1 promoter operated independently of the rest of the MHV-68 genome: eGFP^+^ cells were not necessarily lytic, and neither lytic nor latent cells were necessarily eGFP^+^. In contrast, the expression of eGFP-tagged gM from the endogenous gM promoter correlated closely with ORF65 expression (Fig. 6B, 6C), as is also seen in infected BHK-21 cells [36]. The gM-eGFP virus therefore allowed us to identify lytically infected macrophages.

**Figure 7:**
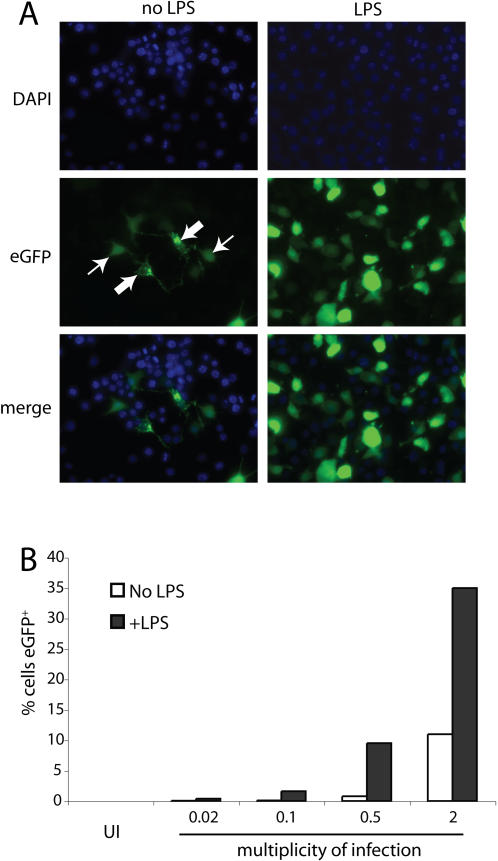
BAC+eGFP and gM-eGFP are differentially affected by LPS. **A.** RAW264.7 cells were infected with BAC^+^gM-eGFP^+^ MHV-68 (2PFU/cell, 18 h), then treated for 4 h with LPS (100 ng/ml). The cells were then fixed and examined for eGFP expression (green). Nuclei were counterstained with DAPI (blue). The thick arrows show gM-eGFP^+^ cells and the thin arrows BAC^+^ eGFP^+^ cells. **B.** RAW264.7 cells were infected with BAC^+^gM-eGFP^+^ MHV-68 at different multiplicities. 18 h later the cells were treated for 4 h with LPS (100 ng/ml). Total eGFP expression (BAC^+^ eGFP plus gM-eGFP) was then quantitated by flow cytometry.

The contrast between BAC^+^ eGFP and gM-eGFP was also evident for a virus that expressed both (Fig. 7). They can be distinguished because gM and gN concentrate in the trans-Golgi network and are excluded from the nucleus [35], [40]-examples are indicated in Figure 7A-whereas free eGFP distributes uniformly. LPS treatment made gM-eGFP difficult to detect and massively increased BAC^+^ eGFP. gM-eGFP may have been partly obscured by the increase in BAC^+^ eGFP, but LPS also reduced gM-eGFP expression (see the eGFP^+^ populations in Fig. 9A). The marked overall increase in total eGFP expression with LPS (Fig. 7B) therefore indicated that many MHV-68-exposed macrophages were infected but gM-eGFP^−^. These were presumably latently infected.

### Functional characterization of MHV-68-infected macrophages

**Figure 8:**
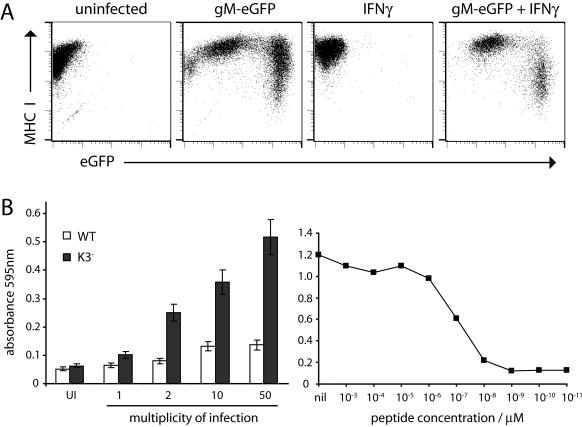
MHV-68 inhibits MHC class I-restricted antigen presentation in macrophages. **A.** RAW264.7 cells were infected overnight with gM-eGFP MHV-68 (2PFU/cell), treated or not with IFNγ (20 U/ml) for a further 24 h, then stained for cell surface MHC class I. The data are from 1 of 2 equivalent experiments. **B.** Peritoneal macrophages from C57BL/6 mice were left uninfected (UI), infected overnight with wild-type (WT) or K3-deficient (K3^−^) MHV-68 at different multiplicities, or incubated with dilutions of the immunodominant H2-D^b^-restricted MHV-68 peptide p56, to relate viral recognition to an equivalent peptide concentration. A p56-specific lacZ^+^ T cell hybridoma was added the next day and β-galactosidase expression assayed by ELISA after a further 20 h. Each bar shows mean±SD results of triplicate cultures. The data are from 1 of 2 equivalent experiments. Similar results were also obtained with bone marrow-derived macrophages.

MHV-68-infected macrophage cultures evidently contain a mixture of uninfected, lytically infected and latently infected cells. Assays of function that fail to distinguish these populations are therefore hard to interpret. We focussed on the lytic (gM-eGFP^hi^) infection phenotype. Flow cytometry established that approximately 50% of lytically infected RAW264.7 cells had down-regulated cell surface MHC class I expression (Fig. 8A). Similar results were obtained for peritoneal macrophages (data not shown). The down-regulation was not reversed by IFN-γ, indeed it became more obvious. This was consistent with K3 function, since the MHV-68 K3 degrades TAP more efficiently in cells treated with IFN-γ [41]. K3 function was confirmed by comparing antigen presentation from peritoneal macrophages infected with wild-type and K3 knockout viruses (Fig. 8B): K3 disruption markedly increased the stimulation of a lytic antigen-specific T cell hybridoma. Comparison with a peptide titration curve suggested that K3 reduced antigen presentation 10-100-fold. The population with intermediate eGFP expression in Fig. 8A presumably derived its gM-eGFP from incoming virions or phagocytosed infected cell debris, and contained both latently infected and uninfected cells (see Figure 7). These showed no evidence of MHC class I down-regulation. Thus, K3 may not be transcribed in latently infected macrophages.

**Figure 9:**
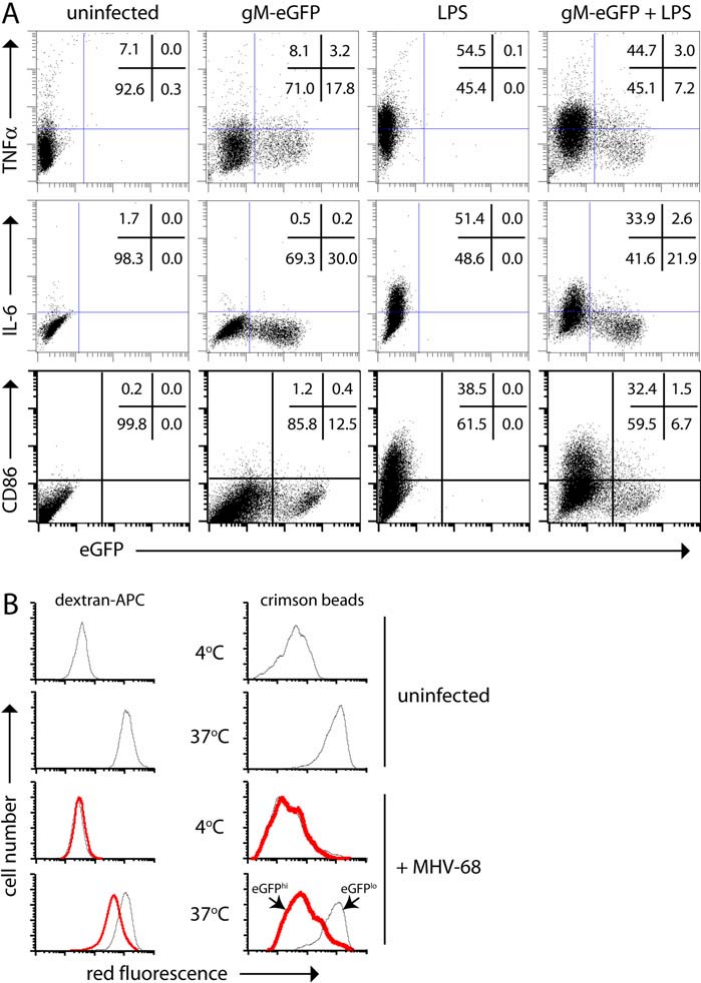
Lytically infected macrophages show multiple functional abnormalities. **A.** Peritoneal macrophages were infected overnight with gM-eGFP MHV-68 (2PFU/cell), treated or not with LPS (100 ng/ml) for a further 6h in the presence of brefeldin-A, then fixed, permeabilized and stained for IL-6 or TNF-α. Cells infected and stimulated the same way, but without brefeldin-A, were stained for CD86 without permeabilization. The data are from 1 of 2 equivalent experiments. **B.** Peritoneal macrophages were left uninfected or infected overnight with gM-eGFP MHV-68 (2PFU/cell), then incubated with 0.2 µm Crimson (625/624) Fluospheres or Alexa 647-conjugated dextran (molecular weight 10 kDa). After 90 min at 4°C (for no endocytosis) or 37°C (for endocytosis) the cells were washed and analyzed by flow cytometry. The infected populations were gated according to high gM-eGFP expression (thick red lines, corresponding to lytic infection) or low gM-eGFP expression (thin black lines, corresponding to no infection or latent infection) and then assayed for red fluorescence. The data are from 1 of 3 equivalent experiments.

Lytically infected RAW264.7 cells showed similar spontaneous TNF-α expression to uninfected cells (Fig. 9A), but less increase in expression with LPS. They also failed to up-regulate IL-6 or CD86. Each function may be specifically inhibited, or lytically infected RAW264.7 cells may no longer respond to LPS. Lytically infected RAW264.7 cells also showed less endocytic uptake of fluorochrome-labelled dextran or 0.2 µm beads (Fig. 9B). Based on LPS-stimulated, BAC eGFP expression, many of the cells in the Fig. 9 cultures would have been latently infected. In contrast to lytic infection, this was not associated with obvious functional abnormalities.

## Discussion

Immune responses generally protect against gamma-herpesvirus-induced disease but fail to prevent viral transmission. Viral inhibition of antigen presentation provides a window of T cell escape [22]. But how do virions evade neutralization by antibody when they are shed from immune hosts? This is different to evading antibody-dependent cytotoxicity within hosts. Indeed, the gammaherpesviruses, which colonize single hosts mainly by latency-associated lymphoproliferation [42], lack the IgG FcR homologs associated with evading antibody-dependent cytotoxicity [43]. Here we have shown that immune sera fail to block FcR-mediated MHV-68 infection. Thus, while the natural antibody response blocks cell binding [27], it evidently fails to inactivate the key process of membrane fusion. Accessory uptake routes such as that provided by FcRs may therefore allow gammaherpes virions to remain infectious when leaving immune hosts.

Virions leaving a persistently infected host or entering a new, naive host traverse complex, dynamic environments. Precisely what infection opportunities and obstacles they encounter are unknown. However, immune hosts shed only small numbers of virions and can have quite high antibody titers, both in serum and mucosal secretions [44]. Exiting virions should therefore meet sufficient antibody to neutralize them if neutralization were efficient. MHV-68 neutralization was clearly limited in scope. Neutralizing mAbs can block fusion [27], [35], but are quite rare in virus carriers and do not work well. Thus, the whole response blocks fusion poorly. We focussed on serum antibody, but the specificities in mucosal secretions are unlikely to be substantially different. Immunoglobulin transport may enrich mucosal secretions for IgA, but the IgA response to MHV-68 is minimal [45] and much of the KSHV-specific antibody in mucosal secretions is IgG [46]. Serum antibody is therefore probably not dissimilar to what an exiting virus would meet.

If epithelial binding is blocked by antibody, antigen sampling and immunoglobulin homeostasis at mucosal surfaces could provide important routes of infection. It might even be considered that the normal infectious particle is an antibody-coated virion. FcRn takes up IgG in adults as well as neonates [33]; FcR^+^ myeloid cells access the mucosal lumen [32]; and M cells provide transcytosis to submucosal FcRs [47]. The fate of incoming virions may therefore depend on their capacity to infect after FcR-mediated uptake. We studied the fate of antibody-coated MHV-68 in macrophages because the destruction of antibody-coated particles is a major part of their function. Antibody-coated MHV-68 escaped this fate. Thus, MHV-68 should be able to escape from the endosomes of other cells too, indeed it normally infects fibroblasts via a low pH-dependent endocytic route [27]. We saw no obvious functional difference between antibody-independent and antibody-dependent macrophage infections. In both cases, MHV-68 remained largely latent for at least 24h, as measured by an absence of lytic gene expression. Latently infected macrophages were functionally indistinguishable from uninfected controls. On switching to lytic infection, MHV-68 inhibited a range of macrophage functions, including MHC class I-restricted antigen presentation, and produced new virions. Thus, FcR-mediated uptake fully restored the infectivity of virions coated with serum antibody. The uptake of antibody-coated viruses by lectins [48] may achieve the same end.

Once MHV-68 has entered a new host, there is no detectable viraemia [49] and viruses lacking gp150, which have a defect in virion release [50], or lacking gL, which have a defect in virion binding [51], appear remarkably normal. Host colonization therefore depends more on cell/cell spread and virus-driven B cell proliferation than on the release of cell-free virions. The capacity of immune serum to damp down lytic replication in this setting probably reflects antibody-dependent cytotoxicity. However, antibody could also divert infection from epithelial cells and fibroblasts, where it is predominantly lytic, to FcR^+^ cells, where it is predominantly latent. This would appear as protection even without an anti-viral effect. Such considerations emphasize the need to understand the molecular mechanisms behind *in vivo* phenomena. These are rarely so straightforward as they first appear.

## Materials and Methods

### Mice and cells

BALB/c and C57BL/6J mice were purchased from Harlan U.K. Ltd. (Bicester, U.K.), housed in the Cambridge University Department of Pathology and infected intranasally with 2×10^4^PFU MHV-68 when 6–8 weeks old (Home Office Project Licence 80/1579). Immune sera were collected at 3–6 months post-infection, by which time the viral load and serum antibody have reached steady state [25], [26]. Macrophages were derived from bone marrow progenitors by culture in RPMI with 10% fetal calf serum, 5% horse serum, 50 µM 2-mercaptoethanol, 2 mM glutamine, 100U/ml penicillin, 100 µg/ml streptomycin, 1 mM pyruvate and 20% L929-conditioned medium. New medium was supplied every 3–4 d and the adherent cells (>95% CD11b^+^F4/80^+^) harvested after 7–14 d. Peritoneal macrophages were obtained by peritoneal lavage of naive mice with Dulbecco's modified Eagle medium plus 5% fetal calf serum. Cells not adherent to tissue culture plates (45 min, 37°C) were discarded. The adherent cells were 80–85% CD11b^+^CD11c^−^F4/80^+^CD19^−^. In flow cytometry-based assays, FSC/SSC gating increased this to >95%. Dendritic cells were grown from bone marrow progenitors in RPMI with 10% fetal calf serum, 50 µM 2-mercaptoethanol, 100 U/ml penicillin, 100 µg/ml streptomycin and 7.5ng/ml GM-CSF. Bone marrow cells were first put on tissue culture plates (30 min, 37°C) and the adherent (macrophage-rich) cells discarded. The culture medium was changed every 2d. After 3d, non-adherent (granulocyte-rich) cells were discarded. After 7d, the non-adherent cells (90% CD11c^+^MHC class II^+^Gr1^−^) were harvested. BHK-21 cells, RAW264.7 cells and 293T cells were grown in Dulbecco's modified Eagle medium with 2 mM glutamine, 100 U/ml penicillin, 100 µg/ml streptomycin and 10% fetal calf serum. Murine embryo fibroblasts were cultured in the same medium plus 50 µM 2-mercaptoethanol.

### Viruses

Infectious MHV-68 was derived from a genomic BAC, which contains eGFP with an HCMV IE-1 promoter as part of a loxP-flanked BAC cassette [35]. Except when eGFP expression was used as a marker of infection (BAC^+^ virus), the BAC cassette was removed by passaging the virus through NIH-3T3-CRE cells [21]. MHV-68 expressing either eGFP fused to the C-terminus of glycoprotein M or eGFP downstream of ORF73 via an IRES have been described [35], [38]. All viruses were grown in BHK-21 cells. Infected cultures were cleared of infected cell debris by low-speed centrifugation (1000×*g*, 3 min). Virions were then concentrated from supernatants by high speed centrifugation (38000×*g*, 90 min). Virus titers were determined by plaque assay on BHK-21 cells [48].

### Flow cytometry

Cells exposed to eGFP^+^ viruses were washed in PBS and analysed directly for green fluorescence. To assay endocytosis, macrophages were incubated with Alexa 647-conjugated 10kDa dextran (Invitrogen) at 100 µg/ml or 0.2 µm Crimson (625/645) Fluospheres (Invitrogen) at 2×10^9^ beads/ml, washed in PBS and analysed for red fluorescence. For surface staining, cells were incubated (1h, 4°C) with MHV-68 glycoprotein-specific mAbs followed by fluorescein-conjugated rabbit anti-mouse IgG pAb (Dako Cytomation) or Alexa 633-conjugated or Alexa 488-conjugated goat anti-mouse IgG pAb (Invitrogen). Fluorescent conjugates of mAbs against CD86, CD11c, CD11b, F4/80, MHC class II or MHC class I were from BD Biosciences. For intracellular cytokine staining, cells were fixed in 1% paraformaldehyde (30 min), permeabilized with 0.1% saponin, and incubated with phycoerythrin-conjugated rat anti-mouse IL-6 or anti-mouse TNF-α mAbs (BD Biociences). For ORF65 staining, cells were permeabilized in 0.1% Tween-20 after fixation and incubated with mAb 12B8 followed by Alexa 633-conjugated goat anti-mouse IgG pAb. Cells were washed with PBS after antibody staining and analysed on a FACS Calibur using Cellquest software (BD Biosciences).

### Immunofluorescence

Adherent cells were washed in PBS, fixed in 2% paraformaldehyde and permeabilized with 0.1% Tween-20. EGFP fluorescence was visualized directly. The MHV-68 ORF65 capsid component was stained with mAb 12B8 [36] plus Alexa 568-conjugated goat anti-mouse IgG pAb (Invitrogen). Nuclei were counterstained with DAPI. Fluorescence was visualized with an Olympus IX70 microscope plus a Retiga 2000R camera line (QImaging).

### Monoclonal antibodies

MHV-68 glycoprotein-specific hybridomas were all derived from MHV-68-infected mice by fusing spleen cells with NS0 cells [52]. Their specificities were determined by flow cytometric analysis of cells infected with wild-type or glycoprotein-mutant viruses, or transfected with recombinant glycoproteins. The mAbs used in this study are listed in Table 1. Isotypes were determined by ELISA (Sigma Chemical Co.). Antibody was concentrated from hybridoma supernatants by ammonium sulfate precipitation and quantitated by ELISA using isotype-matched standards.

**Table 1:** MHV-68 glycoprotein-specific mAbs used in this study.

MAb	target	isotype	reference
T4H7	gp70	IgM	[53]
T2B11	gp70	IgM	[53]
T6G10	gp70	IgM	this study
T3B8	gp70	IgG1	[53]
T1G10	gp70	IgG2a	this study
58-16D2	gp70	IgG2a	[53]
T8H3	gp48	IgG1	[54]
T8A11	gp48	IgG2a	[54]
6D10	gp48	IgG2a	[55]
T6D11	gH	IgG2b	[27]
T2C12	gH/gL	IgG2a	[27]
T7G7	gH/gL	IgG2a	[27]
7E5	gH/gL	IgG2a	[27]
T1A1	gp150	IgG2a	[53]
T4G2	gp150	IgG2a	[54]
T7F5	gp150	IgG2a	this study
T7H9	gB	IgG2a	[56]

### Viral DNA quantitation

DNA was extracted from infected cells and a portion of the MK3 ORF (genomic co-ordinates 24832-25071) amplified by real-time PCR [38]. PCR products were quantitated with Sybr green (Invitrogen) and compared with a standard curve of cloned template. K3 content was normalized by comparison with beta actin, amplified from replicate samples.

### Antigen presentation assay

Peritoneal macrophages from C57BL/6 (H2^b^) mice were infected with wild-type or K3-deficient [21] MHV-68 (2PFU/cell) or incubated with p56 peptide, which corresponds to an immunodominant H2-D^b^-restricted MHV-68 epitope from ORF6 [57]. 18h later a T cell hybridoma was added which produces β-galactosidase in response to p56+H2-D^b^ [59]. After a further 18h, the cells were washed ×1 in PBS and lysed in PBS/5 mM MgCl_2_/1% NP-40/0.15 µM chlorophenol-red-beta-D-galactoside (Merck Biosciences) to assay beta-galactosidase activity. The absorbance at 595nm was read on a Biorad Benchmark Microplate Reader.
